# An introductory review on advanced multifunctional materials

**DOI:** 10.1016/j.heliyon.2023.e18060

**Published:** 2023-07-08

**Authors:** Hani Nasser Abdelhamid

**Affiliations:** a Advanced Multifunctional Materials Laboratory, Chemistry Department-Faculty of Science, Assiut University, Egypt; b Nanotechnology Research Centre (NTRC), The British University in Egypt (BUE), Suez Desert Road, El-Sherouk City, Cairo 11837, Egypt

**Keywords:** Materials, MOFs, Energy, Environmental, Nanomedicine

## Abstract

This review summarizes the applications of some of the advanced materials. It included the synthesis of several nanoparticles such as metal oxide nanoparticles (e.g., Fe_3_O_4_, ZnO, ZrOSO_4_, MoO_3-x_, CuO, AgFeO_2_, Co_3_O_4_, CeO_2_, SiO_2_, and CuFeO_2_); metal hydroxide nanosheets (e.g., Zn_5_(OH)_8_(NO_3_)_2_·2H_2_O, Zn(OH)(NO_3_)·H_2_O, and Zn_5_(OH)_8_(NO_3_)_2_); metallic nanoparticles (Ag, Au, Pd, and Pt); carbon-based nanomaterials (graphene, graphene oxide (GO), graphitic carbon nitride (g-C_3_N_4_), and carbon dots (CDs)); biopolymers (cellulose, nanocellulose, TEMPO-oxidized cellulose nanofibers (TOCNFs), and chitosan); organic polymers (e.g. covalent-organic frameworks (COFs)); and hybrid materials (e.g. metal-organic frameworks (MOFs)). Most of these materials were applied in several fields such as environmental-based technologies (e.g., water remediation, air purification, gas storage), energy (production of hydrogen, dimethyl ether, solar cells, and supercapacitors), and biomedical sectors (sensing, biosensing, cancer therapy, and drug delivery). They can be used as efficient adsorbents and catalysts to remove emerging contaminants e.g., inorganic (i.e., heavy metals) and organic (e.g., dyes, antibiotics, pesticides, and oils in water via adsorption. They can be also used as catalysts for catalytic degradation reactions such as redox reactions of pollutants. They can be used as filters for air purification by capturing carbon dioxide (CO_2_) and volatile organic compounds (VOCs). They can be used for hydrogen production via water splitting, alcohol oxidation, and hydrolysis of NaBH_4_. Nanomedicine for some of these materials was also included being an effective agent as an antibacterial, nanocarrier for drug delivery, and probe for biosensing.

## 1 Introduction

Materials are things that contain a variety of substances [1,2]. The term ‘material’ was coined mainly for solid-state substances that can be used to satisfy the requirement and needs of society. Liquid state substances e.g., petroleum oil/gases should not be considered as materials; because most of these substances are precursors for materials. There are several classes of materials based on strategies including; 1) physical (e.g., solid-state matter or liquid) and chemical properties (organic and inorganic); 2) sources (e.g., natural or synthetic); and 3) biological activity (e.g., biocompatible or toxic; living or non-living). Natural materials may be created from raw materials utilizing a variety of techniques, such as extraction, shape, and purification [3]. On the other hand, there are several ways to produce synthetic materials. Based on the sorts of materials used, humans divided their antiquity into the ages of Stone, Bronze, and Iron. The 19^th^ century, the middle of the 20^th^ century, and the second half of the 20^th^ century are all referred to as the “steel age,” “plastic age,” and “silicon age,” respectively. Materials improved many applications.

This review summarized the literature for several materials and resources. Fe_3_O_4_, ZnO, MoO_3_, CuO, CeO_2_, AgFeO_2_, Co_3_O_4_, SiO_2_, and CuFeO_2_ are examples of metal oxide nanoparticles. Nanomaterials e.g., Ag, Au, Pd, and Pt are examples of metallic nanoparticles. Graphene (G), graphene oxide (GO), reduced graphene oxide (rGO), and carbon dots (CDs) are examples of carbon-based nanomaterials. Biopolymers, such as chitosan, and cellulose, nanocellulose are included. Organic polymers, such as conjugated polymers, and covalent-organic frameworks (COFs) are covered. Other materials such as hybrid materials e.g., metal-organic frameworks (MOFs) are also reviewed.

## 2 Materials: synthesis and characterization

**Fig. 1:**
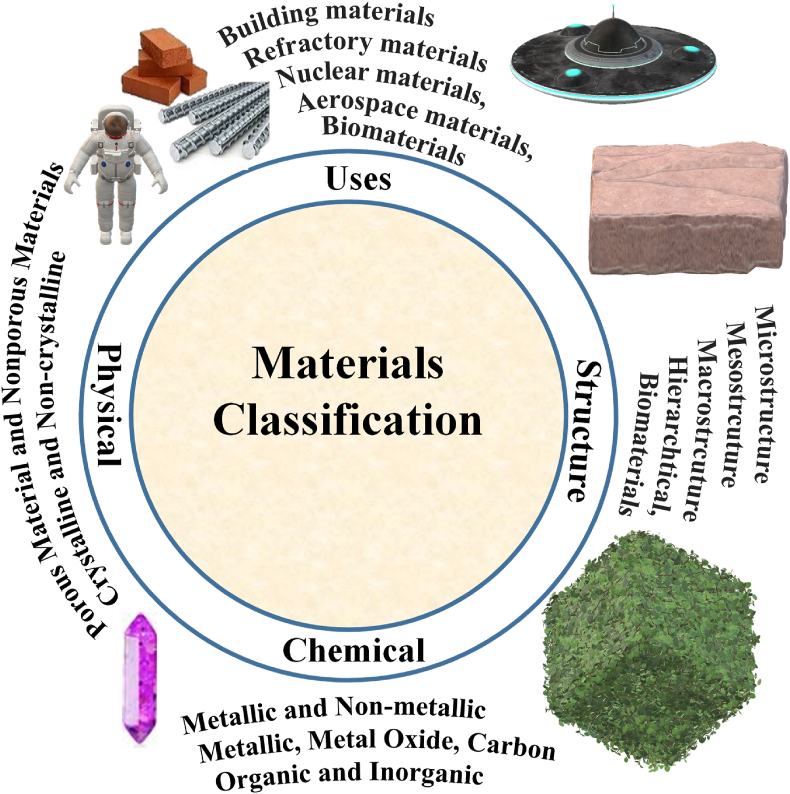
Materials classification.

The m**atter** has been defined as any substance with mass and volume. While **Materials** are terms for objects containing matter or substance. Materials are mainly solid-state substances. Most of the liquid or gas state substrates are precursors for materials. Materials can be classified based on 1) uses; 2) structure; 3) chemical properties; and 4) physical properties (Fig. 1). Based on uses; materials can be classified into building materials (e.g. insulation materials for heat insulation), refractory materials for high-temperature applications), nuclear materials, aerospace materials, and biomaterials. The structure of materials can be evaluated using microscopy or spectroscopy. The materials can be categorized based on the structure into microstructure, mesostructured, macrostructure (large-scale structure), and hierarchical structure. A special type of material was defined as a **metamaterial** for the materials that offer a property that is not found in naturally occurring materials. Materials can be organic or inorganic (Fig. 1). The solid-state solid can be categorized into crystalline and amorphous materials (Fig. 1). The materials can be classified based on the dimensional to 0D, 1D, 2D, and 3D.

Material classification is important. Even though this shows major differences across different groups, there is frequently confusion over the correct taxonomy for a given item. A thin film, for instance, is less than 1 μm thick; but, if the thickness falls below 100 nm, the film may be more appropriately categorized as a two-dimensional (2D) nanomaterial. In the same way that composite materials frequently contain both inorganic and organic components. The term for liquid crystals is best defined as having qualities that are transitional between amorphous and crystalline phases.

**Fig. 2:**
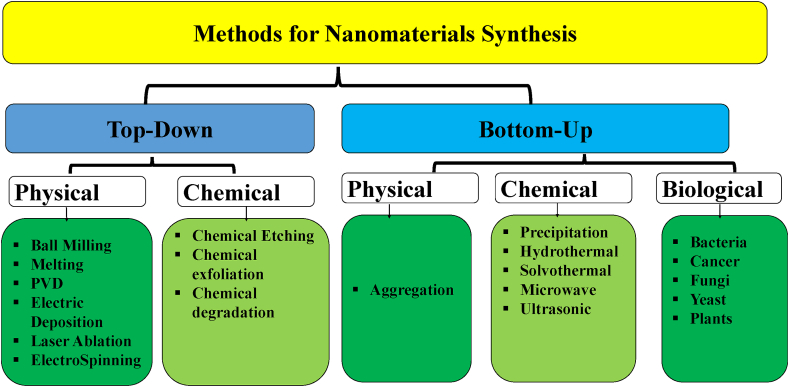
Summary of methods for nanomaterials synthesis with some examples.

Materials can be synthesized using a wide number of methods. Based on size, nanomaterials are the main focus of the current research interests. Nanomaterials are particles with a size below 200 nm. However, some reports call particles with a size less than 1000 nm still define as nanoparticles. Nanomaterials are usually synthesized via two strategies; bottom-up and top-down (Fig. 2). The two approaches aim to decrease particle size via a top-down approach or increase size via bottom-up approaches (Fig. 2). Physical, chemical, and biological methods can be used to achieve this target. The top-down approach uses bulk materials or materials with big sizes. It aims to decrease particle size via physical and chemical methods. Synthesis of nanomaterials is state-of-art. There is no sharp classification between different methods.

**Fig. 3:**
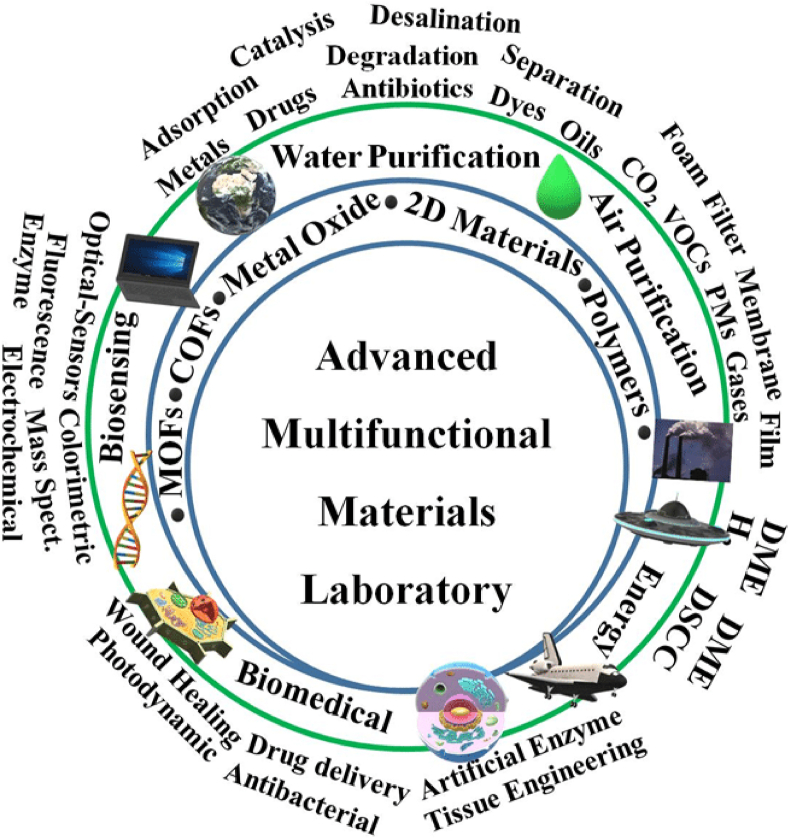
The structure and application reviewed in this review.

The materials should be characterized after synthesis to ensure the successful synthesis of the target materials and confirm their properties. There are a wide number of analytical methods used for materials characterization. The characterization techniques can be classified based on their principles, usage, and information received from their analysis (Fig. 3). Data interpretation is tricky and sometimes misleading. Thus, experience is highly required to avoid misinterpretation. The data interpretation should be supported by other techniques. Most of these techniques can be also used to evaluate the performance of the material for specific applications. The standard recipe for the materials characterization should include the answer to the main questions of the study including these questions; what is the material composition?; what is the material structure, morphology, and particle size?; and what are the physical properties? Material is different from compounds or molecules. Thus, it is a difficult task to fully understand the materials with a limited number of characterization techniques. More techniques are highly desirable to decrease the gap that exists in material characterization. Simply, do more analysis to understand the material's properties.

Table 1 shows most of the characterization techniques used in the literature. It summarized the common techniques that are widely used. We have to admit that there are an unlimited number of analytical techniques. Thus, a short list of the important techniques is included in this review (Table 1). These sections will cover the techniques used for the characterization of the material's composition, phase, purity, structure, particle size and morphology, and porosity. The materials characterization should include qualitative and quantitative analysis.

**Table 1:** Summary of characterization techniques used for materials evaluation.

Information	Type	Techniques	Comments
Composition	Qualitative	EA	C, O, N, S, X analysis, combustion techniques; determining the ratio of elements from within the sample
		MS	Ionization techniques for organic species; give information for molecular weight, and mass per charge (*m*/*z*); Can be extended for particle distribution into a material via imaging technique
	Quantitative	AAF	Measure element in solution as ppm; require digestion with an acid such as nitric acid
		ICP-MS	Sensitive; for liquids; sample pretreatment via acidic dissolution is required
		ICP-OES/AES	
		XRF	Use X-ray for element excitation; can detect ppb
		XPS	For surface analysis with a penetration depth of 10–100 Å
		EDX	Surface analysis technique; Penetration depth of <1000 Å, not suitable for light elements
		TGA	Determine the ratio between components; distinguish among components based on temperature
Structure	Crystal	XRD	SXRD for big crystal; PXRD for powder or small crystal; Avergae of the structure
		SAED	Use for cell parameters determination; 3D diffraction can be achieved using different shots
	Local	EXAFS	Can be used for specific elements in a nanomaterial; require synchrotron facilities
		EELS	TEM-based analysis using electron beam; complementary to EDX
		XPS	Available; local structure of each element in the sample can be obtained via peak deconvolution
	Connectivity	FT-IR	Useful for bond changes or formation
		NMR	Can be used for liquid and solid state
	Inner electronic	EXAFS, EELS, XPS	Determine oxidation state and element valences
Morphology	Light	OM	Maximum magnification 1000x;
	Electron	TEM	Offer 3D model via tomography; can be used for <1 nm;
		SEM	Surface imaging, perfect for morphology, particle size >25 nm
	Probe	SPM	e.g., AFM, STM, and SPE
Size	Solid	TEM	Can be used for particles less than 1 nm; High-resolution TEM can be used for lattice fringes determination
		SEM	Particle size >25 nm; can be used for the determination of 2D thickness
		AFM	Use for the thickness determination of 2D nanomaterials
	liquid	DLS	Measure the light scatters in all directions; Rayleigh scattering; is used only for the particles smaller than the light wavelength (<250 nm).
		LDS	Record the diffraction of a laser beam due to a the interaction with ny particles.
		NTA	Laser techniques; 10–1000 nm; Brownian motion
Optical Properties	Absorption	UV–Vis	Liquid phase; characterize electronic transition within nanomaterials
		DRS	Determine the optical band gap for solid-state nanoparticles
	Emission	FL	Characterize the excitation-emission transition; a useful technique for charge transfer characterization
		PL	Similar to FL; used for particles with long-lifetime emission

**Notes:** Atomic force microscopy, AFM; atomic absorption/emission spectroscopy, AAS/AES; nanoparticle tracking analysis, NTA; STM, scanning tunneling microscopy, STM; Scanning Probe Electrochemistry, SPE; inductively coupled plasma mass spectrometry, ICP-MS; ICP-optical/atomic emission spectrometry (ICP-OES/AES); scanning probe microscopy, SPM.

The chemical composition of nanomaterials is characterized using wide analytical methods. Elemental analysis (EA) is commonly used for the analysis of C, N, O, S, and X (halide, F, Cl, Br, and I). It is called also ‘CHNX’ analysis. It gives usually the ratio of the elements within organic-based nanomaterials with an acceptable standard deviation (SD) of 0.3%. EA using the combustion technique is mainly used for organic compounds. The chemical composition of organic moieties can be characterized using chemical methods such as the sodium fusion test (Lassaigne's test). Techniques such as mass spectrometry (MS) can be used for the chemical composition of organic species directly based on molecular weight via ionization (Table 1). Mass spectrometry can be used for organic and inorganic species with high ionization affinity. The ionization of inorganic species is hard. Thus, powerful techniques are widely used for the ionization of inorganic species via the conversion of the element in the material into the atom. The conversion or ionization of the target element into an atom can be achieved via different methods such as flame atomizers, plasma (temperature of 6000–10000 K), glow discharge atomizers, and others. The composition of inorganic-based nanomaterials is characterized using techniques such as inductively coupled plasma mass spectrometry (ICP-MS), ICP-optical/atomic emission spectrometry (ICP-OES/AES), and atomic absorption/emission spectroscopy (AAS/AES). Most of these methods are used for the determination of the elements in solution. However, solid-state samples can be evaluated via methods such as electrothermal vaporization. The chemical composition of the surface can be determined using techniques such as X-ray photoelectron spectroscopy (XPS), energy dispersive X-ray (EDX), and X-ray fluorescence (XRF). The important aspect of these analyses is the penetration depth of these techniques. EDX and XPS analysis are suitable for the thickness of 2 μm and 10–100 Å, respectively. Surface analysis techniques such as EDX are not suitable for light elements such as C, N, and O. They provide in most cases the ratio between the metals i.e. semi-quantitative analysis. Most of the current techniques display the average of the metal species without respect to the oxidation state of the metal species. Techniques such as thermogravimetric analysis (TGA) can be used to determine the ratio between two different components in composite materials.

The structure of nanomaterials can be evaluated using diffraction techniques such as X-ray diffraction (XRD), and electron diffraction (ED, selected area electron diffraction (SAED). XRD is used for crystalline materials with sharp diffraction peaks at specific Bragg angles. The structure of poor crystalline materials can be solved via the pair distribution function (PDF) using the XRD pattern. XRD requires a big crystal to perform single-crystal XRD (SXRD). Small crystals can be characterized using powder XRD (PXRD). The structure solution of small crystals such as nanoparticles using PXRD is a big challenge due to several reasons such as peaks overlap, and preferred orientation. Thus, techniques such as SAED are used to calculate the unit cell parameters that can be then used to refine the PXRD data for structure solution. XRD offers the structure for the crystal's average. In some cases, the changes in the material take place in the point or plane of the crystal. Thus, it is hard to use XRD for the material characterization. This challenge can be solved using local structure techniques such as Mössbauer spectroscopy, X-ray absorption spectroscopy (XAS, e.g., extended XAS fine structure (EXAFS), electron energy loss spectroscopy (EELS), and X-ray absorption near edge structure (XANES)), and XPS. The connectivity can be also evaluated using techniques such as Fourier transform infrared (FT-IR), and nuclear magnetic resonance (NMR). The inner electronic structure (e.g. oxidation state and the ratio of multi-valence element) of specific atoms in a material can be characterized using techniques such as X-ray fluorescence (XRF), particle-induced X-ray emission, XPS, and Auger electron spectroscopy.

Particle morphology can be determined via imaging using microscopy such as optical light microscopy (OM) or digital microscopy. However, the maximum magnification power of OM is 1000x due to the low resolving power of visible light. Thus, other techniques such as electron microscopy (transmission electron microscopy (TEM) and scanning electron microscopy (SEM)) are widely used for the characterization of small particles such as nanoparticles (1–200 nm). The particle morphology can be determined via a physical probe using scanning probe microscopy (SPM, e.g., atomic force microscopy (AFM), STM, scanning tunneling microscopy (STM), and scanning probe electrochemistry (SPE)). These techniques offer two-dimensional images. However, several shots can be used, and a model can be built for a three-dimensional (3D) image. TEM is the widely used technique for 3D models via TEM tomography.

Particle size is an important parameter for materials characterization. It can be determined using TEM, SEM, AFM, and other microscope techniques. These techniques are used for the solid-state form. Particle size distribution (PSD) can be obtained via the analysis of large particle numbers. The analysis of the size of a particle dispersed in a liquid is determined using different techniques such as dynamic light scattering (DLS), and laser diffraction spectroscopy (LDS). Based on the sources i.e., light or laser, these techniques are named. DLS data is affected by several parameters such as temperature, and concentration. The theory of the DLS analysis is based on the spherical particle. Thus, it isn't reliable data for particles with other morphologies. Other techniques such as nanoparticle tracking analysis (NTA) offer to visualize and analyze particles in liquids following Brownian motion. NTA determines the particle size distribution for a diameter of 10–1000 nm. Both DLS and NTA are based on the Brownian motion. The main difference is in the analysis strategies. NTA is based on video-individual particle positional changes, while, DLS visualizes the total particles using a digital correlator.

The optical properties of nanomaterials are determined using absorption, emission, and scattering techniques. UV–Vis absorption spectroscopy for well-dispersed nanomaterials in liquid or via diffuse reflectance spectroscopy (DRS) in the solid state. These techniques characterize the band gap of the nanomaterials suggesting their potential applications such as photocatalysis. The emission techniques such as fluorescence (FL) and photoluminescence (PL) are the complementary data for absorption techniques. FL and PL are important for the characterization of the emission of the material suggesting the charge transfer within the materials.

## 3 Applications

Our research group can test materials for several applications. Most of these applications are summarized as shown in Fig. 3. The materials were reported for three main areas of research including energy, environmental, and biomedicine. The applications in the energy field can be 1) hydrogen generation via sodium borohydride (NaBH_4_) dehydrogenation, 2) photocatalytic water splitting for hydrogen generation, 3) photocatalytic alcohol oxidation for hydrogen generation and carbonyl compounds synthesis; 4) supercapacitors, and 5) lithium-ion battery. The applications for environmental-based technology can be 1) water treatment via pollutants removal e.g., adsorption and degradation, 2) air purification; removal of greenhouse gases via adsorption, 3) adsorption/photocatalytic oxidation of volatile organic compounds (VOCs), 4) photocatalytic degradation of drugs, antibiotics, and pharmaceuticals, 5) heavy metal removal via adsorption, and 6) precious metal recovery. Biomedical applications can be 1) cancer therapy; chemotherapy, photodynamic, and photothermal, 2) drug delivery, 3) gene delivery using cell-penetrating peptides (CPPs) [4], 4) antimicrobial agents; antibacterial, and antifungal [[5], [6], [7], [8]], 5) nanotoxicity and environmental fate for nanoparticles, 6) bone regeneration, 7) wound healing, 8) tissue engineering, 9) nanozymes and MOFZyme (artificial enzyme based on MOFs materials), 10) biosensing of biomarkers, biological heavy metals, enzymes, and proteins; 11) detection and analysis of pathogenic bacteria; 12) proteomics and clinical research [9]; 13) synthesis of biologically active compounds; and 14) investigate effective matrix for matrix-assisted laser desorption ionization mass spectrometry (MALDI-MS).

Over 80% of industrial processes use catalysis, accounting for $1.5 trillion in yearly global sales, or 35% of the global GDP [10]. The oxidation of organic compounds like alkenes/olefins, alcohol, and dyes is one of several catalytic processes that is crucial for environmental concerns and the production of fine chemicals like medications, paints, and surfactants. The chemical synthesis of useful and valuable compounds depends on the conversion of alcohols by an oxidation process to aldehydes. Additionally, some applications need the catalytic oxidation process, including the oxidation of air pollutants, VOCs, and aqueous pollutants for water treatment. Therefore, the goal of current research is to create a suitable catalyst for the oxidation reaction. Aldehydes, ketones, and other carbonyl compounds are often synthesized using a lot of oxidizing agents like potassium permanganate. However, these oxidants left behind residues that were harmful to the environment. Therefore, more research should be done to identify an appropriate oxidant with minimal adverse effects on the environment. For use in fine chemistry, the noble metal was said to catalyze alcohol oxidation. They have great selectivity and strong catalytic activity. However, they are pricey and need to be used or recycled carefully.

According to the Intergovernmental Panel on Climate Change (IPCC) (https://www.ipcc.ch/), the average world temperature is predicted to climb by 1.9 °C in 2100. According to predictions made by the IPCC, the amount of CO_2_ in the atmosphere will rise from 400 parts per million (ppm) in 2019 to 950 ppm in 2100. The ecosystem and humanity are seriously threatened by the climate's irreversible temperature fluctuations. One of the main contributors to global climate change is the production of gases.

Methane (CH_4_), ethane (C_2_H_6_), and ethene (C_2_H_4_) are examples of condensable organic gases. Inorganic gases, such as hydrogen (H_2_), carbon dioxide (CO_2_), carbon monoxide (CO), nitrogen (N_2_), oxygen (O_2_), and noble gases like He–Kr, are incondensable inorganic gases. Global climatic changes are mostly caused by greenhouse gases like CO_2_ and NOx. One of the main contributors to global warming among these gases is CO_2_ emissions from human activities including breathing, industrial operations, and the burning of fossil fuels. As a result, several techniques, including adsorption and sequestration, were described for CO_2_ capture and utilization (CCU).

Carbon dioxide (CO_2_) levels in the atmosphere have grown by 40% (from 280 ppm to 406 ppm in 1750 and 2017, respectively). According to IPCC's report, atmospheric temperatures may rise by 2 °C in 2036 at the present CO_2_ emission rates. As a result, many methods to lower the amount of human CO_2_ in the atmosphere have been described. It is possible to employ metal-organic frameworks (MOFs) [[11], [12], [13], [14], [15], [16], [17], [18], [19], [20]], including ZIFs for CO_2_ adsorption [21], to mitigate the amount of greenhouse gases in the atmosphere. MOFs have been used as adsorbents and as catalysts for the fixation and photochemical reduction of CO_2_. ZIFs have excellent CO_2_ collection potential. Without experiencing a discernible decline, ZIF-8 demonstrated good selectivity for CO_2_ adsorption over N_2_.

The main greenhouse gas that causes climate change has been identified as carbon dioxide (CO_2_). The sequestration of CO_2_ from the flue gases produced by the burning of fossil fuels has been suggested using a variety of techniques, such as membrane separation, chemical absorption with solvents, and adsorption with solid adsorbents. The creation of a low-cost adsorbent with high selectivity and capacity is necessary for this strategy to be successful. An approach for sequestering carbon that shows promise is carbon dioxide adsorption in the solid-state adsorbent. Adsorption using solid adsorbents is simple to execute in a practical application and needs little energy.

Due to their extreme toxicity and propensity to accumulate in living things, cadmium (Cd) and lead (Pb) are particularly concerning trace heavy metal ions that can contaminate water. They can neither be metabolized nor biodegraded. The neurological, renal, skeletal, nervous, digestive, and reproductive systems can all sustain direct harm from exposure to Cd(II) or Pb(II), in addition to cancer. Over the past few decades, a variety of techniques for removing Cd(II) and Pb(II) have been studied. Adsorption stands out among these techniques because of its beneficial attributes, including its high efficiency, straightforward design, easy regeneration, and cheap operational cost. The need for clean drinking water is increasing exponentially as the world's population grows [22]. Several catalysts were reported for oxidation via advanced oxidation processes (AOPs) [23]. Explore new materials that may advance AOPs for high catalytic performance and better selectivity toward the target molecules. Utilizing nanomaterials, AOPs are the name given to these reactions [24]. The interaction between a catalyst and H_2_O_2_ produces highly oxygen-reactive species (ROS) or hydroxyl radicals (^•^OH). For oxidation through AOPs, a variety of catalysts have been reported []. Investigate novel materials that might improve AOPs for improved selectivity towards the target molecules and higher catalytic performance [25].

Due to their special characteristics and usage in several modern industrial applications, rare earth elements (REEs) recovery is receiving increased attention [19,26]. Significant work has been put into the development of very efficient techniques for the recovery of REEs. For the recovery of REEs, several techniques have been used, including precipitation [27], solvent extraction [28], ionic liquids [29], and adsorption [30]. Adsorption is one of the most efficient methods for separating and recovering various metal ions from aqueous solutions due to its high selectivity, simplicity of use, and environmental friendliness. It is crucial to use the right adsorbents while removing REEs from aqueous solutions. A good adsorbent should be highly recyclable and have a high adsorption capacity.

Hydrogen gas is a good candidate to substitute fossil fuels. Atomic hydrogen includes metal hydrides (MH), such as borohydrides (MBH_4_), as well as molecular hydrogen, which is utilized for pressurized containers and liquid hydrogen tanks. Several techniques were reported to create hydrogen for fuel cells. The first study on the use of the potential of hydrogen as an energy source, was titled “The Hydrogen Economy-An Ultimate Economy?”, and was published in 1972 [31]. Hydrogen has the highest combustion calorific value (1.4 × 10^8^ J/kg) compared to all fossil and biofuels [32].

Due to its high energy density (142 MJ/kg) and ecologically favorable byproduct (water), hydrogen gas is a prospective source of clean energy [33]. Numerous processes, such as water oxidation or splitting [] (electrolysis, thermolysis, and photocatalytic water splitting), as well as the use of phototrophic microorganisms (biohydrogen, BioH_2_) [34], can be used to produce it. To provide hydrogen to end consumers on demand, the hydrolysis of hydrides, such as sodium borohydride (NaBH_4_), appears promising [35]. Direct borohydride fuel cells (DBFCs), unmanned aerial vehicles, and low-temperature fuel-cell applications are among the portable and on-site hydrogen fuel systems that can use this method.

The method is effective and secure for producing hydrogen. At normal temperatures, it produces hydrogen with a comparatively high capacity. NaBH_4_ hydrolysis produces hydrogen with a high purity level and a relatively large hydrogen capacity (10.8 wt%) through a manageable procedure. NaBO_2_, a result of hydrolysis, is another intriguing option for hydrogen storage in solid states. As a result, many heterogeneous and homogeneous catalysts for the hydrolysis and alcoholysis of aqueous NaBH_4_ solution were described [33]. However, the procedure occasionally needs pricey metals like platinum and ruthenium as catalysts. Most modern catalysts are neither stable nor highly efficient. Therefore, a catalyst that can perform several activities at a low cost is essential.

Given the potential of hydrogen gas as a fuel shortly, several manufacturers, including Toyota, Hyundai, and Honda, have lately marketed hydrogen-powered automobiles [32]. For the creation of hydrogen, many techniques have been documented. Hydrolysis of hydrides is one of these ways that is appealing because it has several benefits, including high hydrogen storage (1 mol NaBH_4_ creates 4 mol H_2_), which is significantly more effective than other known hydrogen storage materials. NaBH_4_ is low in weight and volume and generates hydrogen with excellent purity. The technique yields a high rate of hydrogen generation and a high adequate hydrogen storage capacity (10.8 wt%), both of which are adjustable. Although the process is sluggish, a catalyst is needed to speed it up. Thus, many catalysts were suggested for the hydrolysis of NaBH_4_ to liberate hydrogen. Ru, Pt, and Pd, which are pricey precious metal catalysts, are used less frequently as a catalyst for NaBH_4_ hydrolysis, which produces hydrogen. As a result, less costly transition metals have been proposed to take their place. Additionally, to improve the performance of these catalysts, assistance is frequently needed. When compared to alternative supports including MXene and carbon nanotubes (CNTs), ZIF-8 showed superior support for transition metals like cobalt [36]. To produce hydrogen by hydride hydrolysis, many catalysts incorporating ZIFs were reported.

Due to environmental concerns and the scarcity of fossil fuels, there is a discernible interest in creating renewable energy sources. Molecular hydrogen (H_2_) is a prospective renewable source offering a high energy density and power efficiency, among other innovative energy-based technologies [37]. It may be made in many ways, such as via electrolysis-based water splitting or photocatalysis. There are various ways to split water via electrocatalysis and photocatalysis. Photocatalysts have drawbacks including poor catalytic sites and quick recombination of the produced electron-hole (e/h^+^). These semiconductors might be used to create a heterojunction photocatalyst, which could overcome some difficulties.

A possible commercial method for creating hydrogen gas, aldehydes, and ketones is the dehydrogenation of alcohols. For the synthesis of acetone and hydrogen, isopropanol dehydrogenation has promise. The method suggests hydrogen as a potentially clean and viable energy source. Additionally, the other product, acetone, is a crucial chemical reagent for use in both industrial and laboratory settings. It may be utilized as a source of energy [38], as a solvent or reactant in the manufacture of medicines, and as significant chemical reagents such as methyl methacrylate, bisphenol A, vinyl and acrylic resins, lacquers, paints, inks, cosmetics, and varnishes [39].

Drug delivery and gene therapy may help with cancer treatment. Diseases treatment using gene-based therapies has gained significant attention over the past decades. The fundamental idea of gene therapy is to alter or modify defective/missing gene sequences to cure inherited diseases, including cancer [40]. Gene delivery is considered an alternative method to traditional chemotherapy used in treating cancer. However, the transfection of the cell by oligonucleotides (ONs) is tedious due to cell degradation, and low efficiency of cell internalization. Therefore, there is an obvious demand for efficient nucleic acid delivery systems that would ideally promote intracellular delivery. Viral and non-viral vectors were reported as a carrier to improve the cell transfection of oligonucleotides. Among several types of non-viral vectors, cell-penetrating peptides (CPPs), short peptides with sequences less than 30 amino acids, are promising [41]. CPPs show high biocompatibility and offer the potential for large-scale production. However, CPPs exhibit low transfection efficiency [42]. Hybrid conjugation of CPPs with inorganic nanomaterials improved their efficiency and may open new venues for multifunctional treatment [[43], [44], [45]].

### 3.1 Metallic nanoparticles

Metallic nanoparticles e.g., silver (Ag), gold (Au), palladium (Pd), and platinum (Pt), advanced several applications. Silver nanoparticles have been used for many applications such as catalysis, energy, biosensing, MALDI-MS, and mass spectrometry imaging (MSI). In our lab, we investigated Ag NPs' antimicrobial activity against bacterial flora of bull semen [46]. AgFeO_2_ exhibit high antibacterial activity against several bacteria species [47,48]. Ag NPs were used for the freshness determination of fruits and vegetables via graphene-enhanced Raman spectroscopy (GERS) [49]. Silver nanoparticles can be used as a surface for the microextraction of proteins and other analytes via surface-assisted laser desorption-ionization mass spectrometry (SALDI-MS) [50]. It can be also modified with chitosan for the separation and detection of biothiols [51].

The spermicidal effects of Ag NPs against flora bacteria were reported [46]. Silver salts were mixed with melamine. The mixture was then polymerized at 550 °C to generate graphitic carbon-embedded Ag NPs i.e. Ag@C NPs. Analytical techniques such as XRD, XPS, AAFS, TEM, and HR-TEM confirm the material's phases, composition, morphology, and particle size. Ag@C NPs display a particle size of 1–5 nm with an average particle size of 2.5 nm. The nanoparticles were embedded into carbon. Ag@C NPs were investigated as antimicrobial agents in bacteriospermia of fresh semen collected from five fertile bulls. They exhibited high antibacterial activity against bacteria species found in semen. It offered minimum inhibitory concentration (MIC) and minimum bactericidal concentration (MBC) of 3.125–12.5 and 3.125 μg/mL, respectively. There was no detrimental effect (P > 0.05) on the percentage of sperm motility, plasma membrane integrity, acrosome integrity, and normal sperm morphology at concentrations of 15–30 μg/mL. Ag@C NPs is a promising antibiotic agent for bull semen extender during cold storage. It can be used in applications such as the field of artificial insemination [46]. The antibacterial activity of silver ferrite (AgFeO_2_) was investigated. AgFeO_2_ was modified with polyethylene glycols (PEGs) to render their dispersion high [47,48]. The antibacterial activity against pathogenic bacteria was quantified using plate counting, and the turbidity using optical density (OD_600_) at wavelength 600 nm. AgFeO_2_ nanoparticles exhibited high antibacterial activity [47,48].

Silver nanoparticles were modified with 1-octadecanethiol (1-ODT)/4-amino thiophenol (4-AMP) and 1-ODT/1-thioglycerol (1-TG) to prepare Ag@ODT/AMP and Ag@ODT/TG, respectively [50]. The materials were used in microextraction as a pseudo-stationary phase via single-drop microextraction (SDME). They can extract proteins and peptides e.g., insulin, ubiquitin, cytochrome *c*, cysteine, homocysteine, and lysozyme. The separated proteins can be detected after extraction using MALDI-MS. The method can be used for the analysis of real samples e.g., urine and milk [50]. Silver ferrite iron oxide nanoparticles (AgFeO_2_ NPs) were reported for biothiols separation [51]. AgFeO_2_ and AgFeO_2_ modified chitosan (AgFeO_2_@CTS NPs) can be used for the separation of biological thiols e.g., sulfamethizole, thiabendazole, dithiothreitol, and glutathione before the analysis using MALDI-MS and SALDI-MS [51].

**Fig. 4:**
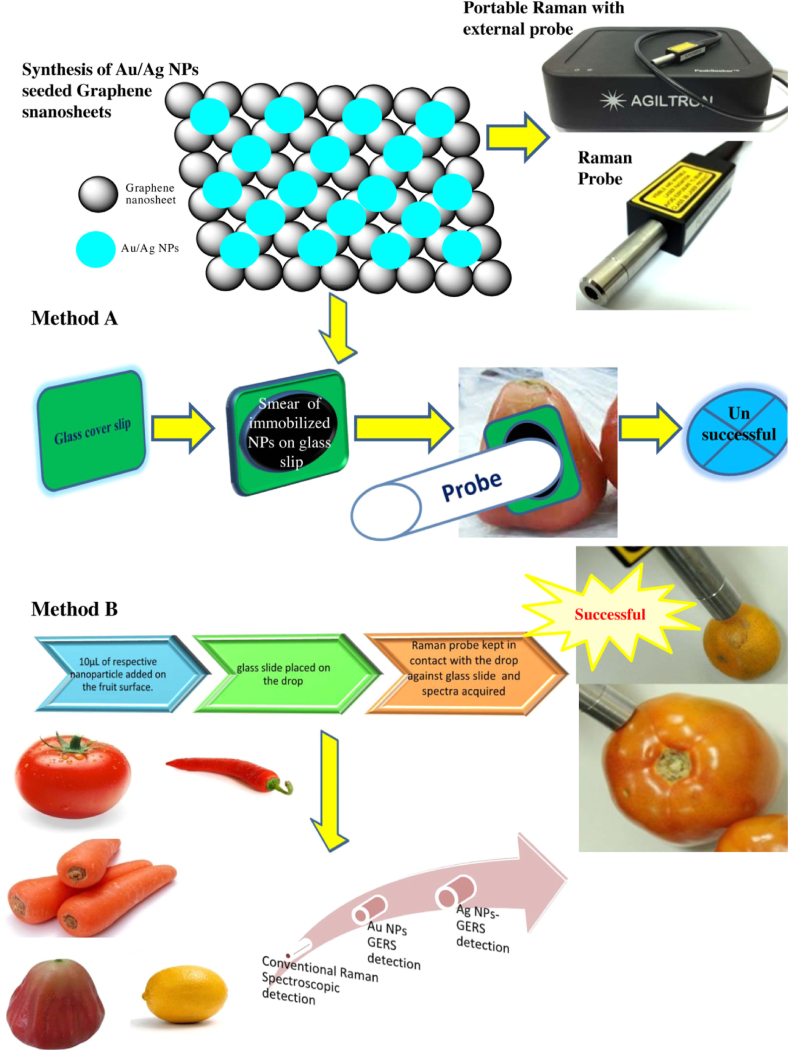
Schematic representation for GERS analysis of fruits and vegetables using Ag and Au NPs. Figure reprinted with permission from Ref. [49].

Au NPs enhanced GERS detection of the fruit's freshness [49]. It can be used as a probe for surface-enhanced Raman spectroscopy (SERS, Fig. 4) using Methods A (Fig. 4A) and B (Fig. 4B). Au or Ag nanoparticles were synthesized into reduced graphene oxide nanosheets (e.g., Au@G and Ag@G). The materials can be used as a probe for the analysis of the freshness of fruits and vegetables (e.g., Carrot, Wax apple, Lemon, Red pepper, and Tomato) [49]. One-pot synthesis of Au NPs@carbon dots was reported for the cytosensing of metals in cancer cells [52]. Au NPs enhanced the analysis of simple molecules to intact cells using SALDI-MS [53].

Noble metal-based catalysts have high catalytic activity for NaBH_4_ dehydrogenation offering high hydrogen generation rates (HGR). Ruthenium-based catalysts were often utilized. Different materials have been used to support noble metal-based catalysts [[54], [55], [56], [57], [58], [59], [60], [61], [62]]. The HGR of the Ru catalyst was higher than that of the Ni_3_B and Co_3_B catalysts, which had HGRs of 1.3 mL/min•g and 6.0 mL/min•g, respectively [63]. Ti_3_C_2_X_2_ (X = OH, F) (a transition metal carbide that resembles graphene and is loaded with Ru) had an HGR of 59.04 × 10^3^ mL/min•g [64]. At room temperature, Ru nanocrystals displayed an HGR of 96.8 × 10^3^ mL/min•g [65]. Additionally, it may be supported by reduced graphene oxide (rGO) via polyvinylpyrrolidone (PVP) stabilization and electrostatic self-assembly [66]. By employing an aqueous ammonia borane solution to reduce Ru^3+^, chitin-supported Ru was created [67]. The HGR for NaBH_4_ hydrolysis at 30 °C was as high as 55.290 × 10^3^ mL/min•g. Noble-based catalysts such as Ru–Ni exhibit good durability and excellent recyclaibility over 300 cycles [68].

Pd has been reported in a variety of forms, including Pd-supported carbon powder (Pd/C) and Pd–C thin films [69]. NaBH_4_ was hydrolyzed using Co_3_O_4_ which had been treated with noble nanoparticles such as Ru, Pt, and Pd [70]. In comparison to Pt–Co_3_O_4_ and Pd–Co_3_O_4_, which displayed HGRs of 4713 mL/min•g and 3445 mL/min•g, respectively, at 25 °C, Ru–Co_3_O_4_ demonstrated the greatest HGR of 6514 mL/min•g. These numbers depend on how much NaOH is present [70]. In comparison to Pt/LiCoO_2_, the HGR utilizing Ru/LiCoO_2_ is somewhat greater [71].

### 3.2 Carbon nanomaterials and their applications

Zero-dimension carbon can be also known as carbon dots (CDs), carbon nanodots (C NDs), or carbon quantum dots (CQDs) [72]. CDs were applied for several promising applications such as drug delivery, imaging, sensing, biosensing, energy-based applications, biomedical, and theranostic [73]. Carbon nanodots, including CDs, graphene quantum dots (GQDs), or CQDs, are emerging new carbon allotropes nanomaterials. Carbon nanomaterials have advanced electrochemical-based applications [74]. CDs have advanced electrochemical applications such as O_2_ and H_2_O_2_ reduction, and biosensing of glucose [75].

C-dots exhibit good optical properties including photoluminescence in the visible range, and high quantum yields (QY). The properties of CDs can be tunable by changing their size, surface modification with functional groups at the graphitic edges of the materials, doping with heteroatoms, or selecting a suitable synthesis method [76]. They can be tuned offering fluorescence emission from blue to green [77]. It has been used for tackling COVID-19 [78], the virus [79]. It offered naked eye sensors [80]. N-doped CDs especially exhibit remarkable acid-evoked fluorescence enhancement under acidic conditions [81].

Two-dimensional carbon nanomaterials such as graphene and GO were intensively used for several applications. Graphene oxide was used for rare-earth metal adsorption [30]. It can be modified with thymine for selective detection of toxic heavy metals such as mercury (Hg(II)) [22]. The layer structure of GO enables the intercalation of an organic matrix such as sinapinic acid [82]. GO can be modified with SiO_2_ for SALDI-MS [83]. It can use for heavy metal detection such as mercury ions [84], lipids [85], and metallodrugs [86]. It exhibited high efficiency for bone and skin wound regeneration [87] and wound healing [88]. It can use for the drug delivery of in-soluble antibiotics such as gramicidin [89]. It can be used as a co-carrier to enhance the gene transfection of CPPs [45]. GO/cellulose nanocomposite accelerated skin wound healing [90]. 2D carbon nanomaterials such as g-C_3_N_4_ and GO were reported for repairing of bone defects in rabbit femurs [91]. Biological analysis of osteogenesis was performed using X-ray, computed tomography (CT), and qPCR analysis. The expression measurement for osteocalcin (OC) and osteopontin (OP) were also included. Based on the data analysis, g-C_3_N_4_ exhibit amount of OC and OP leading to bone defects repairing. Graphene can be used as a surface for SALDI-MS [92].

### 3.3 Metal oxides

Metal oxides such as CeO_2_ enabled the extraction and detection of pathogens proteins [93]. Fe_3_O_4_@SiO_2_ enabled rapid analysis of blood samples offering direct identification of pathogenic bacteria [94]. Magnetic nanoparticles modified graphene oxide was reported for separation and preconcentration of pathogenic bacteria for sensitive detection using MALDI-MS [95]. Chitosan magnetic nanoparticles were reported for endotoxin separation and detection using SALDI-MS [96]. ZnO nanoparticle-modified polymethyl methacrylate (PMMA) was used for dispersive liquid–liquid microextraction for rapid analysis of pathogenic bacteria using MALDI-MS [97]. SnO_2_@GO exhibited high antibacterial activity [98].

**Fig. 5:**
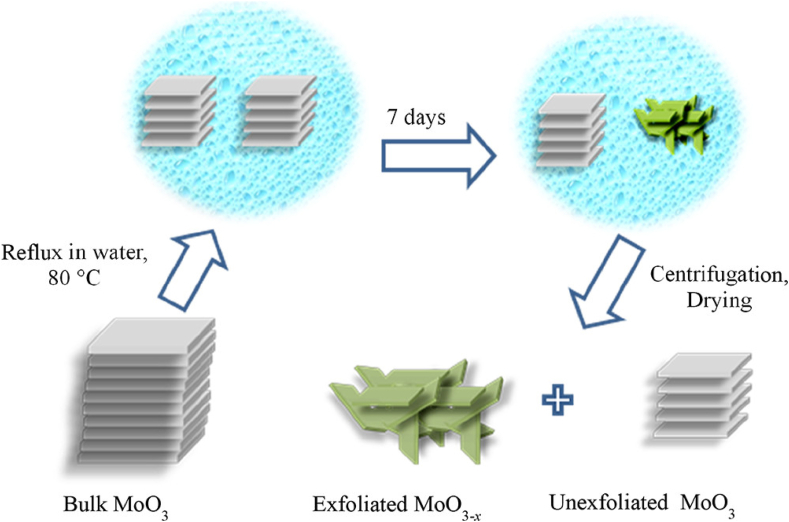
The Exfoliation of α-MoO_3_ into MoO_3–*x*_ Nanosheets. Figure reprinted from Ref. [99]. This is an Open Access Article. Copyright belongs to the American Chemical Society (ACS).

Commercial MoO_3_ was used for the exfoliation to synthesize a few layers of MoO_3-x_ (Fig. 5) [99]. The synthesis procedure involved the reflux of a bulk α-MoO_3_ at 80 °C in water for 7 days. The prepared MoO_3–x_ nanosheets displayed infrared plasmonic properties offering localized surface plasmon resonance (LSPR) peaks at 954 and 1160 nm due to the oxygen vacancies upon light excitation. The plasmonic properties of the nanosheets can be enhanced using visible light irradiation for only 10 min. The materials were used as photocatalysts for dye degradation under visible light irradiation [99].

Ruthenium oxide (RuO_2_) with mesopore was synthesized via a surfactant-assisted procedure [100]. The mesoporous structure of RuO_2_ was achieved using surfactants as a template. The materials exhibited higher catalytic oxidation activity of water using ceric ammonium nitrate (CAN). This is a chemical oxidation of water using CAN as an oxidant.

Magnetic nanoparticles can be synthesized via several procedures including laser techniques [101]. Abdelhamid reviewed the application of delafossite nanoparticles in energy, nanomedicine, and environmental applications [102]. Magnetic nanoparticles of Fe_3_O_4_ were incorporated into polyplexes of CPPs/oligonucleotides (ONs) for cell transfection [44]. Three different oligonucleotides (e.g., plasmid (pGL3), splicing correcting oligonucleotides (SCO), and small interfering RNA (siRNA)) and six CPPs (e.g. PeptFect220 (denoted PF220), PF221, PF222, PF223, PF224, and PF14) were investigated. Magnetic nanoparticles enhanced the cell transfection up to 4-fold compared to the noncovalent PF14–SCO complex, which exhibited higher efficiency compared to a commercial vector called Lipofectamine™2000 [44].

Meta oxides of transition metals can be applied as an effective catalyst for NaBH_4_ dehydrogenation [103]. One of the often-used active metals is cobalt [104]. The hydrolysis of NaBH_4_ is catalyzed by cobalt chloride at a rate that is ten times greater than that of an acid accelerator like boric acid (i.e., Co_2_B). ZnO was synthesized via the sol-gel procedure and was applied as a catalyst for the hydrolysis of NaBH_4_ [105]. The synthesis procedure offers simple modification of ZnO nanoparticles with other metal oxides such as TiO_2_ and CeO_2_ [105].

Metal oxide based on transition metal nanomaterials can be used as effective catalysts and support materials [106]. They exhibited high catalytic performance with low activation energy. Magnetic transition metal oxide can be recyclable after separation simply via an external magnet [107]. They can be synthesized in wide forms including alloy. They offered high HGR [[108], [109], [110], [111], [112], [113], [114], [115], [116], [117], [118], [119], [120]].

### 3.4 Quantum dots (QDs)

Quantum dots (QDs) are nanocrystals with particle sizes <10 nm [121]. Cadmium sulfide (CdS) quantum dots were used for selective biosensing of *Staphylococcus aureus* [122] and proteomics [123,124]. It is a surface for the analysis of several analytes using SALDI-MS [125]. It enabled soft ionization offering the analysis of labile compounds such as metallodrugs [8]. It can also be used for fluorescence spectroscopy [126]. CdS QDs were in-situ grown into chitosan (CTS) enabling CdS QDs@CTS [127,128]. The material CdS@CTS exhibited selective interaction with Cu^2+^ due to the formation of Cd_1-x_Cu_x_S [127,128]. The positive charge on chitosan exhibited also high interaction with the negative charge on the bacteria cell membranes [129]. CdS@CTS was also reported as a carrier for the drug delivery of a natural anticancer drug called sesamol [130].

### 3.5 Biopolymers

Biopolymers including polysaccharides are intensively applied for biomedical applications [[131], [132], [133]]. Polysaccharides were applied as excipients for tablet formulation, dental implants, bone/tissue engineering, and drug delivery [131,132]. They can also be used for antimicrobial textiles [[134], [135], [136]]. Silver ferrite (AgFeO_2_) can be modified with chitosan to render their external surface positive for biothiol separation [51]. Alginate can improve the gene delivery of oligonucleotides [133,137]. Modern technology such as 3D printing enabled simple processing of polylactic acid and hydroxyapatite for water treatment [138].

Anhydroglucose monomer is joined through β-(1–4) bond to form the natural linear-structural biopolymer known as cellulose (C_6_H_10_O_5_)_n_, where n is the degree of polymerization and ranges from 1000 to 5000 depending on the source utilized to extract the cellulose) [[139], [140], [141], [142], [143], [144], [145], [146], [147]]. The most reliable sources of cellulose include plants, seaweeds, sugarcane bagasse, tunicate, marine algae, and bacteria [[148], [149], [150], [151]]. Over a few hundred billion tonnes of cellulose are produced annually. Over time, the market's demand has been rising steadily. High stability in acidic environments, low density or lightweight (density of 1.6 g/cm^3^), and high biodegradability contribute to the excellent mechanical, physical, and chemical properties of cellulose. Cellulose also has good wettability and high tensile strength [[152], [153], [154], [155], [156], [157], [158], [159], [160], [161], [162]]. Thus, cellulose has sophisticated uses in the fields of energy, the environment, and health.

**Fig. 6:**
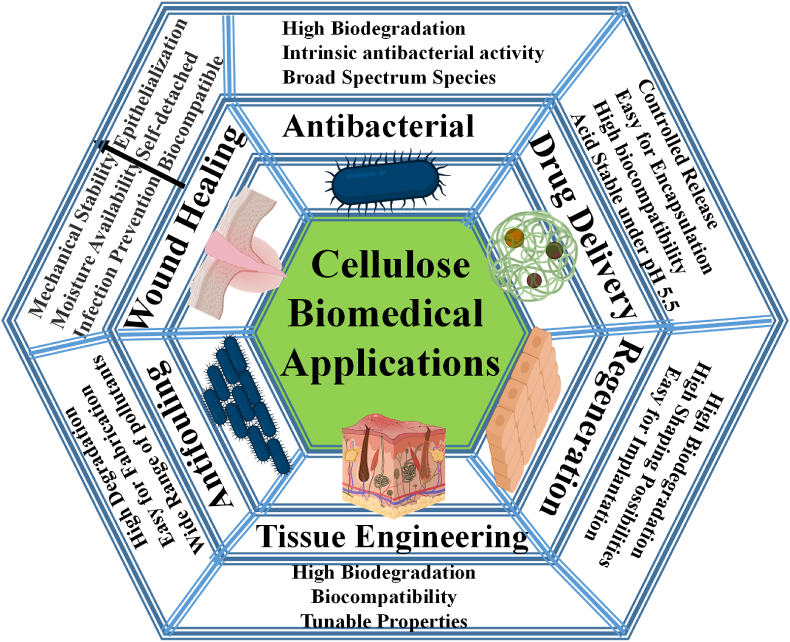
Cellulose-based materials for biomedicine applications. Figure reprinted from Ref. [213], Open Access.

Advanced biomedical uses exist for cellulose-based materials [[163], [164], [165], [166], [167], [168], [169], [170], [171], [172], [173], [174], [175], [176], [177], [178]]. Antibacterial agents [[179], [180], [181], [182], [183], [184]], wound dressing [[185], [186], [187], [188], [189], [190], [191]], medication delivery [160,[192], [193], [194], [195]], tissue engineering [168,177,196], artificial blood vessels [197,198], and UV radiation protection [199,200] were among the applications documented. A variety of materials, including hydrogels [201,202], aerogels [203], membranes [204], and three-dimensional (3D) scaffolds [205,206], may be made from cellulose. They display many traits that make biomedical applications appealing. They provided decent binding qualities [207]. Both organic [208] and inorganic-based compounds can be conjugated with them. Numerous functional groups and substances can alter the surface chemistry of cellulose [[209], [210], [211], [212]]. Recently, Abdelhamid and Mathew summarized the biomedicine applications of cellulose-based materials including drug delivery, tissue engineering, wound healing, antifouling, and antimicrobial agents (Fig. 6) [213].

Unlike other natural biopolymers like cationic chitosan, cellulose has no intrinsic biocidal action [214]. But it may be made into an antibacterial agent in several ways, such as surface modification with other antibacterial agents [181,215].

Investigations on the 2,3-dialdehyde nanofibrillated cellulose's (DANFC) antibacterial efficacy against methicillin-resistant *S. aureus* (MRSA) and *S. aureus* were conducted [216]. The aldehyde groups in DACNF are responsible for the pH reduction (5.7–6.2), which has an antimicrobial impact [216]. Dialdehyde microcrystalline cellulose (DAMC) has also been shown to have antibacterial properties [217].

A wound that was infected with the bacteria *P. aeruginosa* was prevented from growing by a gel of TOCNF (0.2–0.8 wt% in water) [218]. Carboxylate CNF can undergo processing in an autoclave to change its physical, chemical, and antibacterial properties [219]. High antibacterial activity and low toxicity were demonstrated by autoclaved carboxylate CNF towards reconstructed human epidermis (RhE) and L929 murine fibroblasts [219]. *P. aeruginosa* and *S. aureus* were the targets of tests to determine the antibacterial activity of carboxylate CNF with various degrees of oxidation. Compared to non-oxygenated CNF dispersion, oxygenated CNF dispersion displayed greater antibacterial activity.

Pure cellulose nanoparticles' antibacterial properties could be explained by several mechanisms, including a reduction in bacterial cell mobility encircling and trapping the bacteria through the creation of a network, and a lowered pH due to an increase in aldehydes groups in CNFs. It is vital to take into account the existence of alien species since they may generate antibacterial activity through inflammation, such as lipopolysaccharides or endotoxins [220]. The endotoxin level in CNF generated utilizing a modified TEMPO-mediated oxidation process was 45 endotoxin units (EU) per g of cellulose [221]. At low doses, this number might not be harmful. High concentration, however, may need it [222].

Antibiotic-resistant bacteria may benefit from photo-based light radiation therapies [[223], [224], [225], [226]]. They needed the presence of photosensitizer molecules that either generate reactive species (i.e., photodynamic treatment) such as reactive oxygen species (ROS), or absorb light radiation and convert it to heat energy (photothermal therapy). Pure cellulose is devoid of the qualities of the photosensitizer. It is often modified with tiny molecules i.e., photosensitizers, to ensure high absorption of the light. However, they work well to inactivate bacteria when employing low-cost light sources like light-emitting diode (LED) lights [227].

**Fig. 7:**
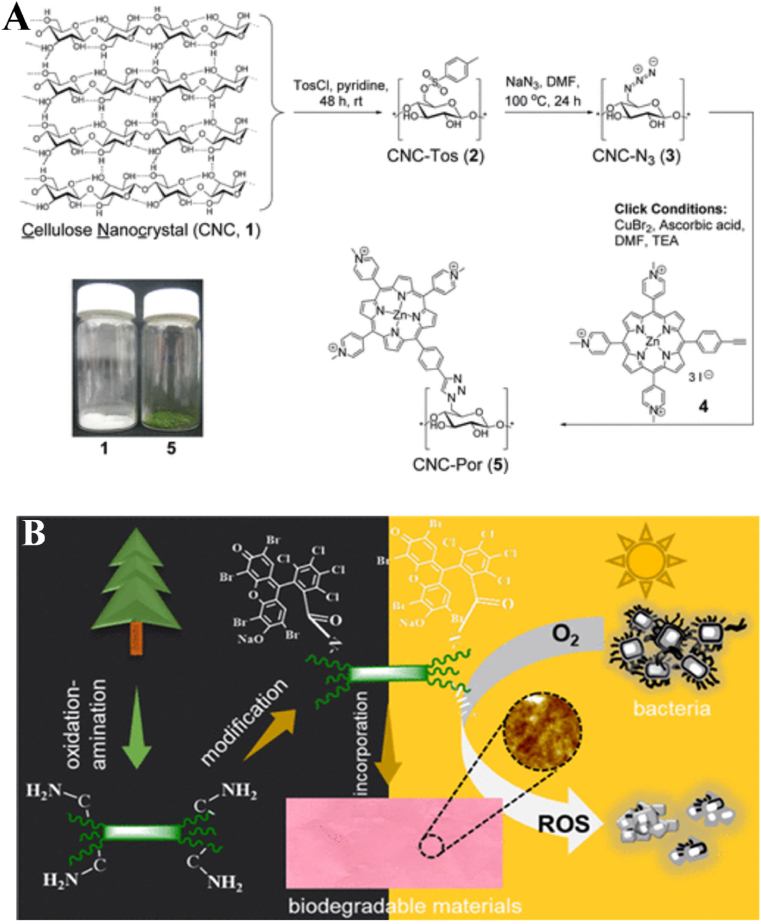
A) Synthesis of CNC-Porphyrine; B) Chemical modification of ANCC with Rose Bengal as photosensitizers. Figure A and B were reprinted with permission Ref. [228], and Ref. [229], respectively. Copyright belongs to the American Chemical Society.

The use of CNC [228] and hairy aminated nanocrystalline cellulose (ANCC) for photodynamical inactivation (PDI) against bacteria has been described [229]. Generation of ROS under light can be achieved via the modification of cellulose with dye molecules. Through the use of Cu(I)-catalyzed Huisgen-Meldal-Sharpless 1,3-dipolar cycloaddition, CNC was chemically changed to form CNC-Por (Fig. 4a). The cellulosic and porphyrinic molecules' respective azide and alkyne groups engage in a reaction (Fig. 4a). Under white light exposure (400–700 nm, 60 mW/cm^2^), the PDI of CNC-Por was studied . More than 99% of bacteria were resistant to the substance (99.9999% for *S. aureus*) . Rose bengal (RB), a naturally occurring photosensitizer, was used to modify ANCC through a covalent link (Fig. 7A–B) [229]. For the pathogens, Listeria monocytogenes and S. Typhimurium, RB-ANCC demonstrated PDI over 80% when exposed to natural light (Fig. 7B). It's interesting to note that ANCC increased the free RB's PDI against *S. Typhimurium* [229].

For scalable antibacterial treatment utilizing PDI, cationic porphyrin (Por(+)) conjugated cellulose was used as paper []. Por(+)–the modified cellulosic paper was irradiated for 30 min. *Acinetobacter baumannii*, *P. aeruginosa*, *Klebsiella pneumoniae*, *vancomycin-resistant Enterococcus faecium* (VER), *S. aureus*, and other bacteria and viruses were tested for antibacterial and antiviral efficacies. For every species that was looked at, such as bacteria and viruses, the inactivation efficiencies were more than 99.9% [223].

One benefit of PDI utilizing cellulose-based materials is a high antibacterial efficacy of up to 99.999% [223]. The technique can be used to treat germs that are resistant to antibiotics. With relative efficiencies of 99.995%, 99.5%, and 99%, photosensitizer-conjugated cellulose fibers may be employed to inactivate viruses such as the dengue-1 virus, influenza A, and human adenovirus-5 [539]. The creation of materials like paper [223], fibers [230], or textiles [231] using cellulose chemistry enables scalable and simple applications for antibacterial treatment. With the aid of cutting-edge techniques like photo-strain-triggered click ligation [232], it offers instant covalent modification. It may provide photoactive textiles with a new market [233]. Cellulose nanocrystals (CNCs) were reported as immune modulators [234].

**Fig. 8:**
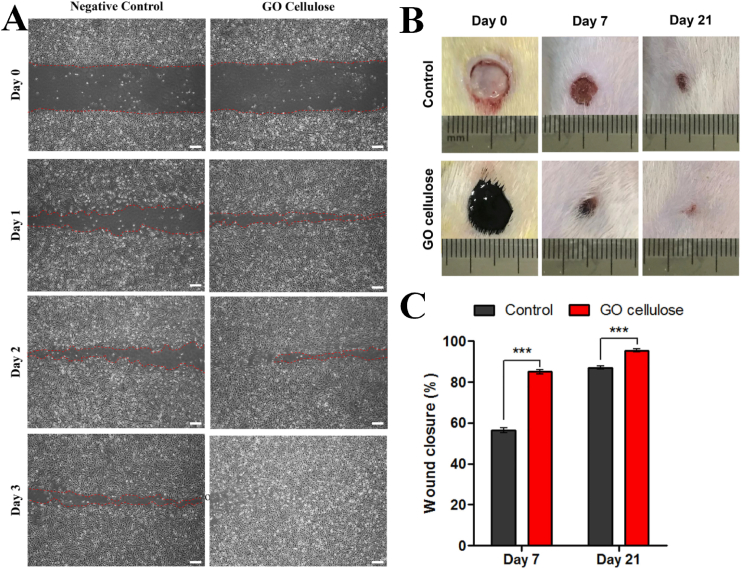
Biological acidity of GO/cellulose using A) cell migration, Scale bar = 200 μm, B) the skin wound, and C) The percentage of wound closure, Figure reprinted with permission from Ref. [90]. Copyright belongs to Elsevier (2021).

The high antibacterial activity of cellulose can be achieved using inorganic-based antimicrobial agents [[235], [236]]. Antibacterial agents made of silver, including silver sulfadiazine (SSD), is often utilized. The preparation of BC/SSD involved ultrasonically impregnating SSD into the BC membrane [237]. Significant antibacterial activity was demonstrated by the BC/SSD membrane against many microorganisms, including P*. aeruginosa, E. coli*, and *S. aureus* [237]. High biocompatibility was shown by the membrane [237]. The dispersion of GO was enhanced by methylcellulose [90]. The cytocompatibility of EA.hy926 human endothelial cells (ECs) demonstrated good biocompatibility. Using an induced wound scratch model for EA.hy926 ECs, the cell migration under the influence of GO-cellulose was revealed (Fig. 8A–C). The cell migration was increased by GO-Cellulose (Fig. 8A). Rats with 8 mm-diameter full-thickness wounds on their dorsum were used to test the in-vivo wound healing process [90].

To dress wounds, cellulose has various merits. It is inexpensive to do. Using inexpensive materials like wood, it may be made into useful structures like membranes [185]. When used as a wound dressing, cellulose-based membrane performs better than commercial porous regenerating membrane [500]. When compared to Suprathel® (a commercial lactocapromer-based wound dressing), the epithelialization of a wood-based dressing like NFC demonstrated quicker healing [187]. Compared to traditional synthetic fiber dressings, BC dressings are less expensive [238].

Numerous techniques, including cross-linking with chemicals based on silanes, can be used to alter the surface characteristics of cellulose-based wound materials [239]. For femoral artery and liver damage models, the material was examined for wounds. Organosilane's chemical alteration created a hydrophobic barrier that prevented blood from penetrating (blood loss was less than 50%) and hastened the blood clotting process. It provided both models with a brief period of hemostasis [239]. The cellulose nanoparticles' high surface charges improved protein adsorption and may encourage cell adhesion.

Since the cellulose-based dressing is often transparent, wound therapy may be assessed without removing or replacing it [185]. Due to the many hydroxyl groups prevalent in the cellulose structure, cellulose-based membranes adhere well to wet wound surfaces with no evidence of allergic or inflammatory reactions [185]. Compared to commercially available wound-healing dressing, cellulose-based dressing enables quicker self-detachment. They are effective in treating infected wounds [240]. Third-degree burn wound healing can be managed using thymol-enriched BC hydrogel [241].

Drug delivery has progressed thanks to cellulose-based polymers [195,[242], [243], [244], [245], [246]]. To provide multifunctional applications, they can be coupled with nanomaterials like magnetic nanoparticles (MNPs) [247]. Folic acid surface modification of cellulose promotes selective cell absorption and binding via a cellular mechanism controlled by the folate receptor [248,249]. For the administration of hydrophobic medicines including docetaxel, paclitaxel (PTX), and etoposide, cellulose serves as an efficient drug carrier [250].

**Fig. 9:**
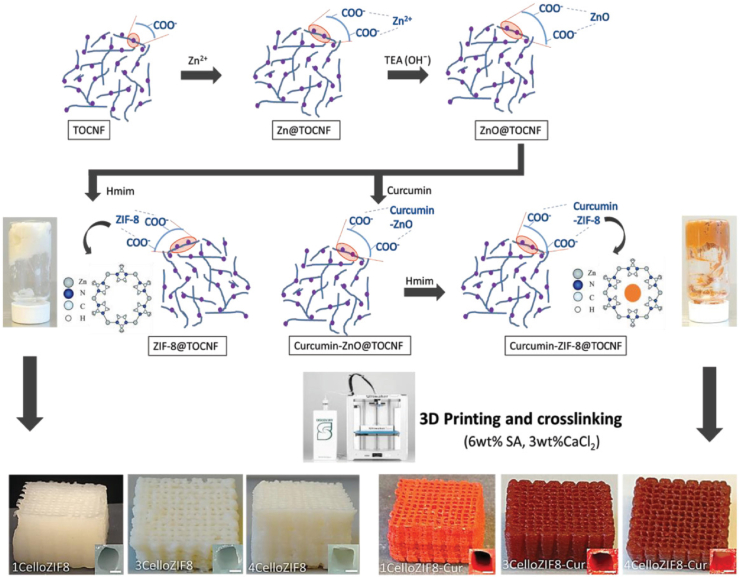
In preparation and 3D printing of cellulose-ZIF8. Figure reprinted with permission from Ref. [252]. Copyright belongs to John Wiley & Sons (2019).

**Fig. 10:**
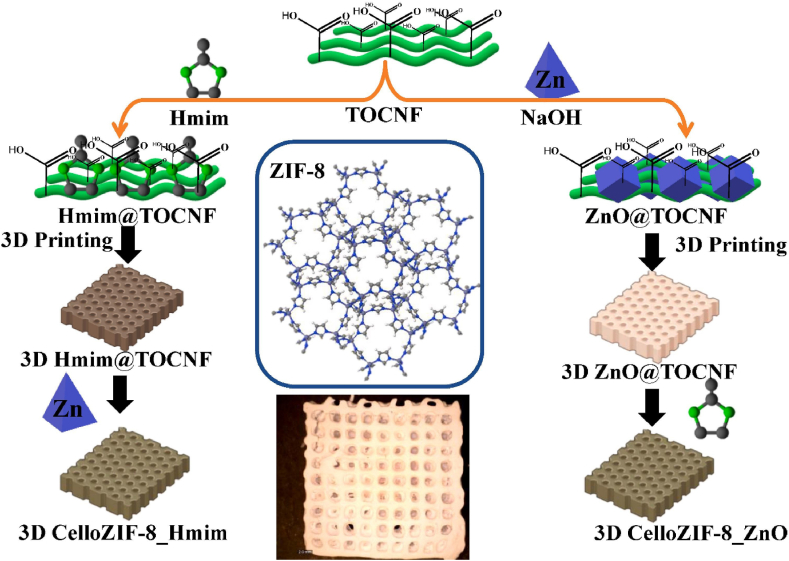
3D printing procedure of CelloZIF-8 using a binder-free procedure. Figure reprinted from the Open Access Ref. [253].

Curcumin (CUR) treatment for prostate cancer cells was made more effective by hydroxypropyl methylcellulose [251]. Comparing CUR alone to CUR-conjugated cellulose, substantial apoptotic alterations were observed. Comparing cellulose to other carriers, cellulose likewise demonstrated the maximum cellular absorption [251]. For the medication delivery of CUR, TOCNF, and MOFs were utilized (Fig. 9) [252]. The composite of TOCNF/ZIF-8 allowed 3D printing to transform the material into a 3D network [252]. MOF powder may be printed using cellulose. In-situ synthesis uses it as a template and binder for MOFs. Under physiological pH (5.5), the materials can release the CUR medicine [252]. A simple procedure of 3D printing of cellulose/ZIF-8 was also reported using a binder-free procedure (Fig. 10) [253]. The loading of ZIF-8 can reach 70 wt%. The printed materials can be used as adsorbents and catalysts for water treatment of organic pollutants. They can be also adsorbed with heavy transition metals with high adsorption capacities. They were also applied as a filter for CO_2_ adsorption [253].

**Fig. 11:**
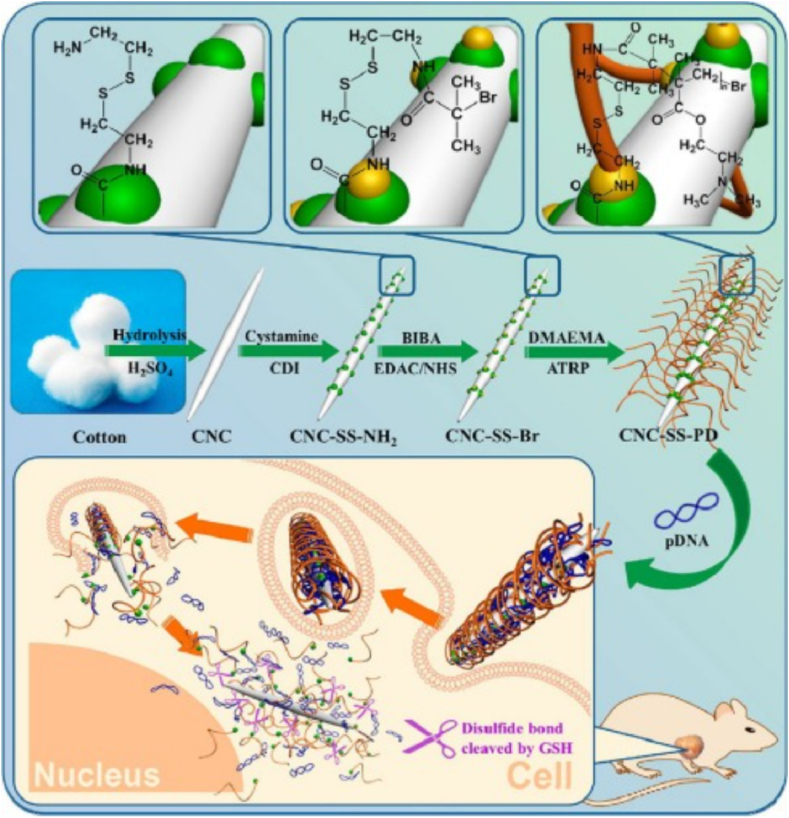
The preparation of CNC-SS-PD and their use for the gene delivery process. Figure reprinted with permission from Ref. [257]. Copyright belongs to ACS (2015).

Gene delivery of oligonucleotides like siRNA was also accomplished using cellulose-based materials [254,255] and chitosan-based materials [256]. You may think of them as non-viral vectors . To deliver pDNA, modified CNCs were reported (Fig. 11A–F). The disulfide (SS) bond is formed during the polymerization process to produce CNC-SS-PDs (Fig. 11A–F) [257]. The CNC-SS-PDs demonstrated high transfection effectiveness with minimal cytotoxicity. Non-viral gene delivery vectors are promised by cellulose-based materials [[258], [259], [260]] .

**Fig. 12:**
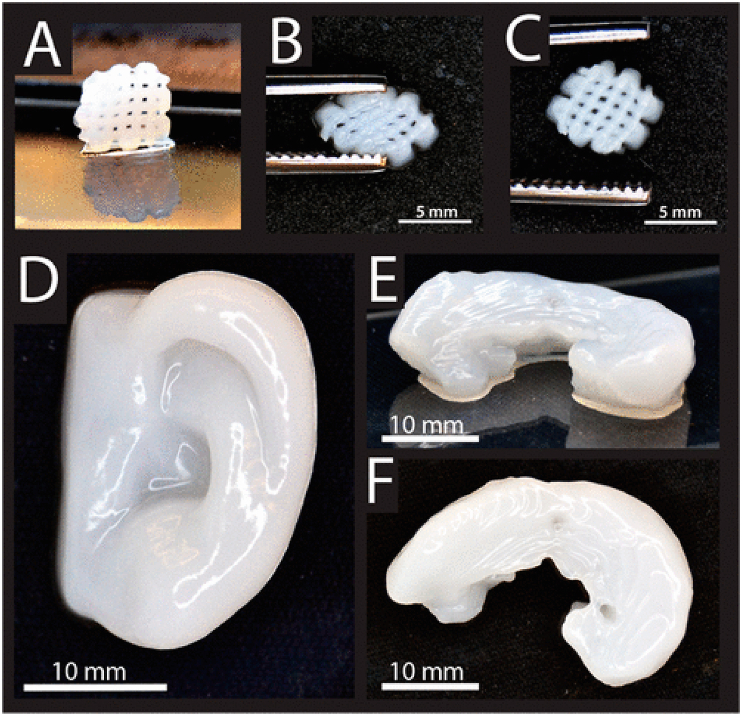
3D printing of cellulose/alginate into A-C) small grids (7.2 mm × 7.2 mm), D-F) 3D printed human ear in different views. Figure reprinted with permission from Ref. [261]. Copyright belongs to ACS (2015).

For medication delivery, cellulose-based materials provide many benefits [[262], [263], [264], [265], [266], [267], [268], [269], [270], [271], [272], [273], [274]]. They can be utilized to release medicines that are hydrophobic, ionizable, water-soluble, and insoluble [250,275]. They make it possible to give two medications simultaneously [276]. Locally tailored medication release with long-lasting qualities was made possible using CNC hydrogels [277,278]. Without the use of gelatin, cellulose can be produced as capsules [279]. It can be used to give medications orally [280]. It is possible to model cellulose-based hydrogels' drug delivery under pH- and temperature-responsive conditions. Cellulose and other biopolymers such as alginate can be 3D printed into cartilage structures such as a human ear and sheep meniscus (Figure 12) [261]. They can be also used for bone regeneration [281,282].

Cellulose-based advanced several applications such as biomedicine including antifouling [162,213,283,284]. They improved bioengineering [285] and water treatment via pollutants adsorption [152]. Cellulose/ZIF-8 composite was used for water remediation via adsorption and degradation of organic pollutants such as dyes [161]. Cellulose enabled three-dimensional printing of porous materials such as leaf-like zeolitic imidazolate frameworks (ZIF-L), denoted as CelloZIF-L. Direct ink writing (DIW) or robocasting was used to proceed with the materials. The materials with a ZIF content of 84% were achieved. The materials were used for the adsorption of carbon dioxide (CO_2_) and heavy metals offering capacities of 0.64–1.15 mmol/g (at 1 bar, 0 °C) and 554.8 ± 15 mg/g, respectively. The adsorbent exhibited selectivity toward Fe^3+^, Al^3+^, Co^2+^, Cu^2+^, Na^+^, and Ca^2+^ of 86.8%, 6.7%, 2.4%, 0.93%, 0.61%, and 0.19%, respectively [286]. Cellulose enabled also the processing of ZIF materials into filter paper [287,288] and foams [289]. Most of these biopolymers are biodegradable compared to synthetic polymers [290]. They can proceed into the membrane for oil separation [291].

Chitosan improved gene delivery [256]. It can stabilize magnetic nanoparticles that enabled high-cell transfection [43]. Magnetic nanoparticles modified chitosan was used for surfactant capture and analysis using SALDI-MS [292]. Chitosan can be modified with thymine to enable mercury (II) preconcentration for SELDI-MS analysis [293]. It can be used as a porogen for creating mesopores inside microporous materials [259]. The created hierarchical porous materials can be then used for oligonucleotide delivery offering efficient gene treatment. Chitosan mitigates the toxicity of CdS QDs offering efficient drug delivery of the anticancer drug sesamol [130].

### 3.6 Metal-organic frameworks (MOFs)

Materials that are organic-inorganic crystalline and porous are known as MOFs [[294], [295], [296], [297], [298], [299], [300], [301], [302], [303], [304], [305], [306], [307], [308], [309], [310], [311], [312], [313], [314], [315], [316], [317], [318], [319], [320], [321], [322], [323], [324], [325], [326], [327], [328], [329], [330], [331], [332], [333], [334], [335], [336]]. They have low density (0.2–1 g/cm^3^), large specific surface areas (>10,000 m^2^/g in some cases), and well-defined pore structures with high porosity up to 50% of the crystal volume [337]. Reticular synthesis can be used to create them, resulting in framework structures or organized networks with solid connections connecting organic and organic moieties [338]. By constructing secondary building units (SBUs) with appropriate organic linkers, the construction networks between the two moieties can adjust the geometry of MOFs [339]. MOFs can have their functional groups and porosity altered using techniques like post-synthetic modification (PSM) [340]. It is also possible to create multivariate MOFs (MTV-MOFs) with various metal nodes or clusters and additional organic functions [341]. Strong bonding between the moieties in many MOF materials provides for exceptional chemical and thermal stability in the 250 °C to 500 °C temperature range [342]. For applications like the gas adsorption of CO_2_ from the atmosphere or hot-flue gases, MOFs' excellent chemical stability, particularly against water molecules, is often necessary [92]. MOFs were applied for several applications, including chemical conversion/fixation of CO_2_ [[343], [344], [345], [346], [347], [348], [349], [350], [351], [352]], catalysis [[353], [354], [355]], photovoltaic devices [356], sensors [357], theranostic platform [358], hydrogen production [[359], [360]] , dye sensitizing solar cells (DSSCs) [310], osmotic power generators [361], and other [362].

MOFs were reported for treating contaminated water. High surface area, high porosity, and chemical and thermal stability are typical features of MOFs. It has been reported that MOFs have been investigated for the removal of heavy metal ions from aqueous solutions. The necessity for a filter or centrifuge to extract MOFs following the adsorption process makes large-scale application of MOFs still difficult. For its extensive uses, it will be crucial to design and create readily separable MOFs from aqueous solutions while overcoming the drawbacks of current adsorbents.

MOFs are porous materials that self-assemble and have a variety of topologies, large surface areas, and customizable pore structures [337,363]. A subclass of microporous MOFs known as zeolitic imidazolate frameworks-8 (ZIF-8) is made up of 2-methylimidazole (Hmim) as an organic linker and zinc metal ions as coordination centers [[364], [365]]. Microporous ZIF-8 has attracted a lot of research interest for heavy metal adsorption because of its desirable properties such as high surface area, high chemical and thermal stability, adjustable pore structure, and ease of synthesis [[366], [367], [368], [369]]. However, the limited applicability of microporous ZIF-8 is due to its small pore size (3.4 and 11.4) and lack of an acido-basic site . This challenge can be solved using hierarchical porous ZIF crystals [[370], [371]].

MOFs advanced several applications including biosensing . Lanthanide MOF was reported for the detection of ferric ions and vitamin C [19]. The material was stable and can form high dispersion with high fluorescence emission signals. Fe(III) ions can selectively quench the fluorescence signal enabling a linear relationship in the concentration range of 16.6–167 μM with a limit of detection (LOD) of 16.6 μM (S/N ratio of >3) [19]. Explosive materials such as nitroaromatic were detected using Zn-MOF [20].

A bimetallic of ZIF-8 (Co, Zn)‐ZIF-8 and semiconductor photocatalyst TiO_2_ (Co@ZIF‐8/TiO_2_) was reported for water splitting and hydrogen generation [372]. Co@ZIF‐8/TiO_2_ showed a photocatalytic HGR of 13 mmol•h^−1^•g^−1^ representing a 151‐fold high catalytic performance of pristine TiO_2_ [372]. Co@ZIF‐8 improved also hydrogen generation via the hydrolysis of NaBH_4_ [373]. Carbonized MOF enabled selective dehydrogenation of isopropanol [362].

We reported several procedures to prepare hierarchical porous zeolitic imidazolate frameworks (ZIFs) [312,374]. Template-free and template-based procedures were reported [375]. Dye encapsulation and one-pot synthesis of hierarchical porous (microporous–mesoporous) ZIF-8 were reported for CO_2_ sorption and adenosine triphosphate biosensing [376]. A cobalt ZIF material, ZIF-67, enhanced the hydrogen release from NaBH_4_ [303,377]. The generated hydrogen can be used for dye degradation [377]. ZIFs-based materials were reviewed as efficient adsorbents and catalysts for CO_2_. They can be applied to convert CO_2_ gases into value-added compounds [[378], [379]]. ZIF-8 and ZIF-67 can be in-situ grown into cellulosic filter paper that was used as an efficient catalyst for the reduction of water pollutants such as nitrophenols [154]. Our synthesis procedures offered several advantages including the formation of a hierarchical porous structure with fast and potential to use for large-scale production [319].

ZIFs materials including ZIF-8 are biocompatible materials [306]. Thus, ZIF-8 was widely used for biomedical applications [304] including gene delivery [258]. However, our recent study showed the transfer of the metal ions into the environment that caused a significant effect on shaded outdoor mice carrions [380]. A zirconium-based MOF, UiO-66, can enhance bone generation offering induction treatment of bone defects in rabbit femoral condyles [381]. UiO-66 catalyzed the hydrogen formation via the hydrolysis of NaBH_4_ [311]. It was also reported as a precursor for the synthesis of ZrOSO_4_@C for hydrogen generation [360] and dimethyl ether formation [14].

A cerium MOF (Ce-MOF) exhibited Fenton-like properties that enabled oxidation the of olefins and alcohol. It can also be used for dye degradation [382]. It offered 100% and 53% conversion of cinnamyl alcohol and styrene, respectively. It provided high selectivity of 75% and 100% towards styrene oxide and benzaldehyde, respectively. It can catalytically degrade organic pollutants such as dyes [382]. Ce-MOF was also used probe for the fluorescence detection of ferric ions and hydrogen peroxide [357].

A copper-based MOF (Cu and 1,4-benzene dicarboxylic acid as metal nodes and linker, respectively) [[383], [384]] was in-situ grown into the fiber of cotton textile via a solvothermal procedure [75]. CuBDC@Textile was investigated as a solid sensor and adsorbent for volatile organic compounds (VOCs). It offered selective detection of pyridine via the colorimetric method. Pyridine turned the turquoise color of the prepared materials into deep blue color. It offered a pyridine adsorption capacity of 137.9 mg/g [75]. Lanthanide MOFs were also incorporated into cotton textiles for the photodegradation of stains for smart textiles [18].

**Fig. 13:**
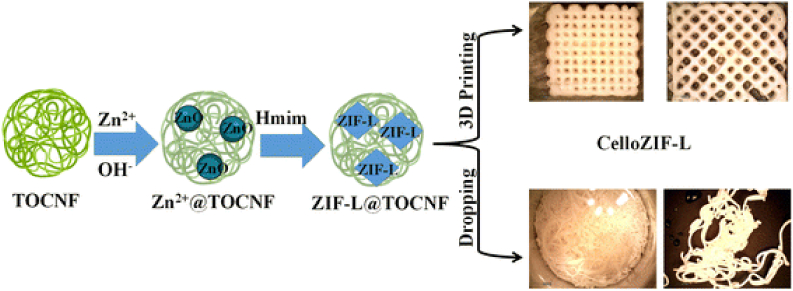
Schematic representation for the synthesis of ZIF-L in TEMPO-oxidized cellulose nanofibers (TOCNF) and 3D printing into cubes and filaments. Figure reprinted with permission from Ref. [286].

Three-dimensional (3D) printing can be used to proceed MOF materials such as ZIF-L into 3D objects with custom porosity and dimension (Fig. 13) [286]. DIW was used to proceed with the materials. The printed materials with a ZIF content of 84% were achieved. The materials can adsorb CO_2_ and heavy metals. 3D CelloZIF-L exhibited adsorption capacities of 0.64–1.15 mmol/g for CO_2_ gases at 1 bar (0 °C). They showed adsorption capacities of 389.8–554.8 mg/g for Cu^2+^ ions with a selectivity of 86.8% toward Fe^3+^ ions [286]. A filter paper containing cellulose and ZIF-8 were reported [287,288]. The prepared filter paper, denoted as CelloZIFPaper, was used for heavy metal adsorption (66.2–354.0 mg/g). CelloZIFPaper was also tested as a flexible electrode for toxic heavy metal detection [287,288]. The reader can directly go to our recent Review on the topic of cellulose-MOF composite (denoted as CelloMOF) and their applications [301]. CelloMOF enabled multifunctional applications being efficient adsorbents and catalysts [161]. ZIF-8 was also reported for the recovery of rare-earth elements [309].

Magnetic nanoparticle-modified MOF materials were reported for heavy metal adsorption and removal [300]. Fe_3_O_4_@ZIF-8 and Fe_3_O_4_@UiO-66–NH_2_) were investigated for the adsorption of Cd^2+^ and Pb^2+^ ions. Fe_3_O_4_@UiO-66–NH_2_ and Fe_3_O_4_@ZIF-8 offered adsorption capacities of 714.3 mg/g, and 370 mg/g for Cd^2+^, respectively, and 833.3 mg/g, and 666.7 mg/g for Pb^2+^, respectively [300].

CuBDC was applied for the conversion of nitrophenol into aminophenol [334]. It was also used for the synthesis of CuO-embedded C i.e. CuO@C [15,383]. CuO@C exhibits a particle size of 36–123 nm [383]. It can be used as an antifungal agent against *Alternaria alternata*, *Fusarium oxysporum*, *Penicillium digitatum*, and *Rhizopus oryzae* with inhibition zones of 36, 20.2, 16, and 10.2 mm, respectively [383]. CuO@C was also used as a photocatalyst for pharmaceuticals e.g. paracetamol degradation [384]. It offered an efficiency of 95% within 60 min [384]. It can also be used for the reduction of 4-nitrophenol into 4-aminophenol [333]. CuO@C undergoes catalytic degradation of organic dyes via the in-situ generated hydrogen [314].

ZIF-67 was carbonized into Co_3_O_4_@N-doped C [385]. The materials after carbonization were used as electroactive material for electrode fabrication. Co_3_O_4_@N-doped C electrode offered a specific capacitance of 709 F/g at 1 A/g [385]. It can be also used as a co-catalyst to enhance the photocatalytic water splitting of semiconductor TiO_2_ [359]. ZnO@C was prepared via carbonization of ZIF-8 [386]. It was used as a supercapacitor [386]. ZIF-8 was used to prepare a ZnO@C photocatalyst that can degrade dyes [299,387]. ZnO@C can be also used as an efficient catalyst for methanol dehydration forming dimethyl ether that can be used as energy fuel [302].

### 3.7 Covalent organic frameworks (COFs)

COFs were used as support for the *in-situ* growth of palladium nanocrystals (Pd NCs@COF) [388,389]. Pd NCs@COF was used as the catalyst for carbon-carbon coupling reactions with high efficiency and excellent selectivity [388,389]. A composite of COFs material with two-dimensional nanoparticles e.g., graphene oxide, boron nitride, and graphitic carbon nitride (g-C_3_N_4_) was synthesized via a one-pot procedure [315]. The nanocomposites were used in water treatment via organic pollutants adsorption [315].

**Fig. 14:**
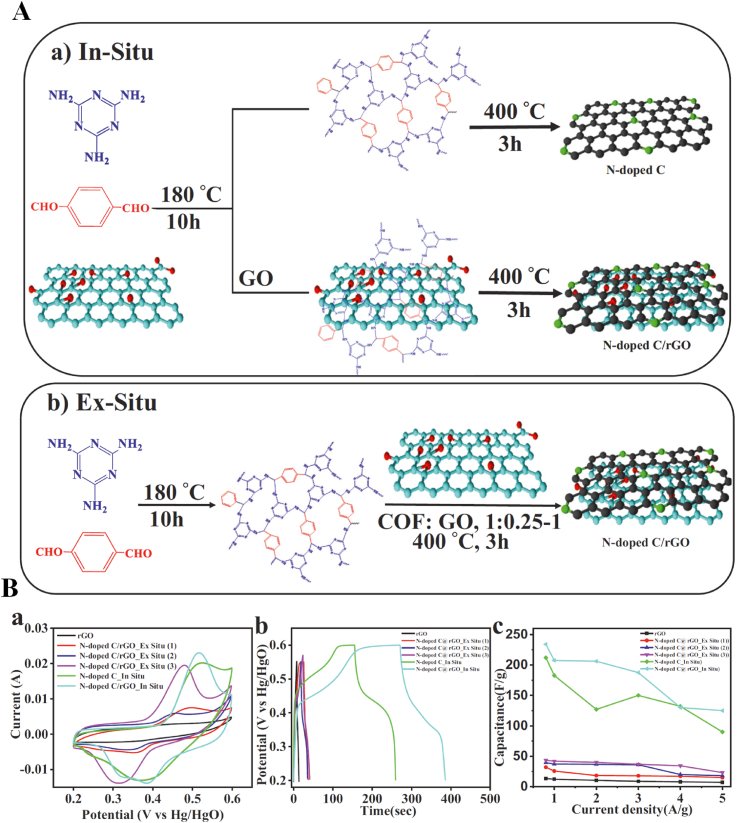
A) Synthesis procedure of the materials and B) electrochemical performance of the prepared electrode using a) CV curves at 50 mV/s scan rates b) GCD curves, and c) capacitance over current density. Figure reprinted with permission from Ref. [392].

COFs have an advanced energy sector [390,391]. A triazine COF was synthesized via *in-situ* (Fig. 14A) and *ex-situ* (Fig. 14B) procedures in the presence of graphene oxide (GO, Fig. 14) [392,393]. The composite was used to produce N-doped C/rGO after carbonization. N-doped C/rGO displayed a specific capacitance of 234 F/g at the current density of 0.8 A/g. The electrochemical performance of two symmetric supercapacitor devices displayed specific energy and specific power of 14.6 W h kg^−1^ and 400 W kg^−1^, respectively (Fig. 14) [392]. A one-pot synthesis of COFs/graphitic carbon nitride (g-C_3_N_4_) nanocomposite was also reported in our lab [,^394^]. The synthesis procedure involved the polycondensation of melamine and benzene-1,3,5-tricarboxyaldehyde in the presence of g-C_3_N_4_. COF/g-C_3_N_4_ was used as a precursor for the synthesis of N-doped carbon and N-doped carbon/g-C_3_N_4_. The prepared materials were used for supercapacitors and lithium-ion batteries (LIBs). COF, COF/g-C_3_N_4_, N-doped carbon, and N-doped carbon/g-C_3_N_4_ exhibited specific capacitance of 211, 257.5, 450, and 835.2 F/g, respectively. N-doped carbon/g-C_3_N_4_ was used to assemble asymmetric devices that offered energy density and power density of 45.97 Wh·kg^−1^ and 659.3 W kg^−1^, respectively [,^394^].

## 4 Conclusions

A summary was reported for materials and their applications in several fields such as environmental trends e.g., water remediation, air purification, and gas storage; energy e.g., production of hydrogen, dimethyl ether, solar cells, and supercapacitors; and biomedical sectors e.g., sensing/biosensing, cancer therapy, and drug delivery. Effective adsorbents and catalysts for the treatment of new water pollutants can be synthesized. Catalysts for the breakdown of pollutants, the synthesis of new organic compounds, and the reduction/oxidation of organic pollutants are all possible with these materials. Adsorption of greenhouse gases including CO_2_ and VOCs makes them useful as filters for air cleaning. They may be put to work splitting water, oxidizing alcohol, and hydrolyzing NaBH_4_ to generate hydrogen. Antibacterial, drug delivery and biosensing are only a few of the biomedical uses for them.

## Author contribution statement

The author has significantly contributed to the development and the writing of this article.

## Data availability statement

Data will be made available on request.

## Declaration of competing interest

The authors declare that they have no known competing financial interests or personal relationships that could have appeared to influence the work reported in this paper.
